# Numerous Paragraphs of Interest

**Published:** 1895-06

**Authors:** 


### Medical News and Miscellany.

---

**Married,** at Dallas, Texas, May 20 (ult.), Dr. John D. Wingate, of Ennis, Texas, to Miss Annie Louise Lindsley, of Dallas.

---

**Dr. W. Y. Gadberry,** of Yazoo City, Mississippi, died May 12, ult., aged about 70. Dr. Gadberry was one of the ablest, most original and most eminent physicians in Mississippi.

---

**Dr. W. E. Pugh,** of Rockport, has been appointed Quarantine Officer at Aransas Pass, vice Dr. J. D. Westerwelt resigned, because of reduction in salary, and further reduction by reason of discount on State warrants.

---

**Our** friend, Dr. B. D. Smith, of St. Elmo, Texas, has associated with himself in the practice Dr. W. E. Holtzclaw, formerly of Atlanta, Georgia, more recently of Buda, Texas. They have the best wishes of the JOURNAL.

---

**The Doctors** are in it. Our good-looking friend Dr. A. W. Fly has been re-elected mayor of Galveston, and the handsome Prof. H. A. West, Secretary Texas State Medical Association, has been chosen alderman for his ward. Now, look out for the "fair round belly with fat capon lined."

---

**Died,** May 24 (ult.), at his father's residence, after a brief illness, Ralph Smith, son of Dr. Q. C. Smith, of this city, age sixteen. Ralph was a bright and promising lad. He was a pupil at the high school and would have graduated soon. The JOURNAL extends its sympathy to the bereaved family.

---

**Kola Nuts.**—Frederick Stearns & Co., who are direct importers of the fresh (undried) nuts of the kola, that wonderful plant that is now attracting so much attention, will send two of the nuts, free of charge to any physician who will request it, mentioning the Red Back. They suggest the nuts may be planted. Literature on the plant and its medicinal virtues, will be mailed with the nuts, if desired.

**Prof. of Pharmacy.**—The Regents of the University of Texas, at a recent meeting in Galveston, examined the credentials of quite a number of applicants for the position of Professor of Pharmacy in the School of Pharmacy of the Medical Department of the University of Texas, which position was made vacant by the death, in April, of Prof. James Kennedy. Some of the applicants were men of national distinction; one a graduate of the Johns-Hopkins University, and a choice where so many were excellent, was hard to make. It fell, however, to Mr. I. M. Cline, of Houston, a gentleman who for many years has been connected with the U. S. Signal Service, or Weather Bureau.

---

**The Sensation of the Day.**—"Coin's Financial School" gives the facts about gold and silver in a simple, clear and most forcible manner, so that the dullest intellect can not fail to grasp it. It does it in a pleasing style, free from the dry tediousness which usually attends such subjects. You devour it with eager pleasure and want for more. Its revelations are astounding, and we predict it will either right things or cause trouble.

The recital of the wrongs inflicted upon the industrial classes and the poor by our government in its dubious course, financially, especially upon the farmer, is enough to excite violent indignation, and if redress is not speedy and complete, we fear it will incite to insurrection. The writer as good as says, that demonetization of silver in 1873 was procured by English gold in England's interest, and is responsible for the evils we are suffering; and that the worst has not come.

We will send a copy of the 50 cents edition *free* to *every one* who sends us $2.00 for a year's subscription,—whether new subscriber, old subscribers renewing, or delinquent remitting on account.

This book should be read by every free-born American citizen who pays taxes to the government. This offer is good for thirty days only.

---

**The North Texas Medical Association** will meet in Bonham, Texas, Tuesday, Wednesday and Thursday, June 18th, 19th and 20th, inst.

The following, with an attractive programme, has been issued:

VAN ALSTYNE, TEXAS, May 30th, 1895.

DEAR DOCTOR:—The next semi-annual meeting of the North Texas Medical Association will be held in Bonham, beginning at 2 o'clock, Tuesday, June 18th, 1895.

These meetings are most enjoyable socially, and are the source of much valuable information that can be gathered in no other way. It is sincerely hoped there will be a full attendance of the membership, and that all regular physicians in good standing, who would keep abreast with progressive medicine, will be present.

The cordial welcome that awaits us, and the unusually full and varied programme, should appeal to all to come, bringing their choicest thoughts and most valuable experiences for mutual exchange.

S. D. MOORE, M. D., President.

T. M. TAYLOR, M. D., Secretary.

---

**Sanitary Climatology.**—Circular No. 4, of the Weather Bureau, containing information relative to the investigation of climate on health, previously noted in the *Journal*, furnishes blank forms of the report desired. They seem to be simple, compact and yet sufficiently comprehensive. Supplies of the forms and of the blank envelope may be obtained by those interested, on application to the Bureau. It is intended to collate the vital statistics thus obtained with the meteorological statistics by general averages and by particular and selected events, as the comparison of the general mortality with the average conditions of the weather for the week, and the passage of storms and cold or hot waves, the appearance of epidemics, etc. Also, in instances of well-defined weather disturbances, comparisons of vital and meteorological statistics will be made by daily periods. For example, a storm appearing in the western part of the country, will be followed day by day, as it passes eastward across the country, and the illness and deaths reported for these days from the localities traversed will be compiled and compared with the same kinds of facts reported both before and after the storm. The same plan will be pursued in dealing with hot and cold waves. By these methods it is hoped to give in time, definite information as to how and how much the accidental and constant variations of the weather affect the sick and well, and in what way the present forecasts and weather charts can be used in both curative and preventive medicine.—*Journal A. M. A.*

---

**The twenty-first annual meeting** of the Mississippi Valley Medical Association will occur in Detroit, Mich., September 3, 4, 5 and 6, 1895. This Association is now in a more prosperous condition than ever before. The membership list shows a large increase annually, and the character of the scientific work accomplished at each meeting is of the very highest. The officers and committee of arrangements are working unceasingly for the success of the Detroit meeting, and, although early, indications are that a meeting of unusual size and interest will be held in September. The profession of Detroit are united in their efforts to have the gathering in their city outshine all previous ones. The social features of the meeting will leave nothing to be desired in that direction.

A meeting of the officers, committee of arrangements and auxiliary executive committee was held in St. Louis in April. The prominent railroads were all represented at this meeting, and the railroad officials present promised a united effort to obtain a half-fare rate to Detroit.

It was decided to make the annual address a special feature of the meeting. September was chosen as the time of the meeting for two reasons. First, because the medical colleges will not have opened, and opportunity will thus be given those connected with these institutions to be present; second, because this is the most delightful time of the year in which to visit the beautiful city of Detroit.

A cordial invitation is hereby extended to you by the executive committee to be present. Titles of papers should be presented to the secretary as early as possible.

FREDERICK C. WOODBURN, Secretary.

**American Medical Publishers.**—This Association held its second annual meeting at the Eutaw house, Baltimore, on the 6th and 7th of May, with the following in attendance:

Dr. J. C. Culbertson, Cincinnati, Ohio; Miss Dora Jones, St. Louis, Mo.; Dr. John C. Le Grand, Anniston, Alabama; Dr. C. F. Taylor, William B. Saunders, Philadelphia, Pa.; Miss Hackedorn, Toledo, Ohio; Dr. F. E. Stewart, Detroit, Mich.; J. MacDonald, Jr., Irving J. Benjamin, Dr. Ferdinand King, Dr. P. H. Fairchild, New York City; Dr. R. W. Lowe, Bridgeport, Conn.; Dr. W. C. Wile, Danbury, Conn.; Dr. H. M. Simmons, Dr. William B. Canfield, Baltimore, Md.; H. A. Mathie, Dr. A. H. Ohman-Dumesnil, Dr. I. N. Love, St. Louis, Mo.; Dr. Landon B. Edwards, Richmond, Va.; Dr. S. E. Hudson, Austin, Texas; Dr. Wm. F. Bartlett, Philadelphia; Dr. T. D. Crothers, Hartford, Conn.; Dr. Gilbert I. Cullen, Cincinnati, Ohio; Dr. Henry S. Upson, Cleveland, Ohio; Dr. E. E. Holt, Portland Maine; J. M. Grosvenor, Jr., Boston; Charles Wood Fassett, St. Joseph, Mo.

Nineteen new members were admitted and questions of the day affecting medical publishers were profitably discussed.

Beginning with July 1st, a monthly bulletin will be issued for the benefit of members of the Association. It is to be edited by Drs. P. H. Fairchild, J. MacDonald, Jr., and Ferdinand King, New York City; Dr. J. C. Le Grand, of Anniston, Ala., and Charles Wood Fassett, of St. Joseph, Mo.

The Secretary was authorized to issue in pocket form, a revised list of medical advertisers.

Upon invitation, the Association banqueted with the Medical Editors, on Monday evening.

The officers re-elected were as follows: President, Dr. Landon B. Edwards, of Richmond, Va ; Vice-President, Dr. J. C. Culbertson, Cincinnati, Ohio; Treasurer, J. MacDonald, Jr., New York City; Secretary, Charles Wood Fassett, St. Joseph, Mo.; Dr. J. C. Le Grand and Irving J. Benjamin were elected on the Executive Board.

---

**The United States Public Health Department.**—For some years past it has been a prominent aim of the American Medical Association to cecure for the United States a central Government Department of Public Health, and preliminary steps have from time to time been taken to memorialize Congress to create such Department, together with a State paid Medical Secretary. Once again the Association brings the matter forward in the form of a memorandum to the Senate and House of Represenlatives, prepared by the Chairman, Dr. C. G. Comegys; and appended to this document is a draft bill to give effect to the object referred to. It is admitted that much excellent work is performed through the agency of different medical departments connected with the army, the navy, and different scientific bodies; but it is contended that government can, in a wider way, promote the public welfare hy creating a Department of Public Health, the head of which should be a physician, a member of the Cabinet, and on a position of equality with the heads of the government departments. Under section 2 of the proposed bill the work of the department is set out. It may be regarded as covering all that is done by the Medical Department of the Local Government Board in this country, by the Registrars-General's Department, and by the Factory Department of the Home Office; and it is intended to be provided with the machinery for compiling information for the purposes of the various State and other bodies on a multitude of social and health subjects, including the questions of food supplies, intemperance, prostitution, elementary education, etc. . . . . .

With regard to the organization of the Board, we believe the Association is right in demanding that its head and its executive officers should be medical men. Indeed, we have heard only one valid reason for a change in the same direction in this country. It is said that if a new department in public health were created it would be the youngest department in the State and would have to rank as such. But the Local Government Board, which has public health functions in addition to a multitude of other duties, is gradually becoming a Ministry of the Interior, and it is assumed that when its poiition as such in the State is fully recognized, it will probably absorb one or two functions still performed by other departments, and its chief will be raised to the position of a Secretary of State. This is the ambition of the secondary State departments in this country, and there are those on the permanent staff of the Local Government Board who would resent the severance from the board of its public health functions, for such severance would diminish its importance and cut adrift a staff which, to say the least, has brought them some credit in the eyes of the public. In the States no such considerations as these arise, and it is to be hoped that the new department, when created, will be mainly in the hands of those who best know what is wanted for the control and the improvement of the health of the public.—*Brit. Med. Journal*, May 4, 1895.

